# Primary Gastric and Duodenal Mucosa-Associated Lymphoid Tissue Lymphoma With Symptomatic Anemia

**DOI:** 10.14309/crj.0000000000001438

**Published:** 2024-07-17

**Authors:** Abdulla Alabed, Faisal Abubaker, Omar Sharif, Alddana Zayed, Eman Aljufairi

**Affiliations:** 1 Internal Medicine Department, King Hamad University Hospital, Busaiteen, Bahrain; 2 Endoscopy Department, King Hamad University Hospital, Busaiteen, Bahrain; 3 Pathology Department, King Hamad University Hospital, Busaiteen, Bahrain

**Keywords:** gastroinestinal lymphoma, duodenal MATL lymphoma, *H.pylori*

## Abstract

Mucosal-associated lymphoid tissue (MALT) is a low-grade lymphoma derived from marginal zone B cells in extranodal tissue. Gastric MALT lymphoma is frequently seen; however, duodenal MALT lymphoma is rare, and there is no standardized knowledge up to date about the management of the disease. We present a case of a 56-year-old woman with gastric and duodenal MALT lymphoma.

## INTRODUCTION

The most common extranodal primary site for non-Hodgkin lymphoma is the gastrointestinal tract. Gastrointestinal lymphomas include several histologic subtypes, and mucosal-associated lymphoid tissue (MALT) lymphoma is the most common histological type, accounting for 40% of gastrointestinal lymphomas.^1^ MALT lymphoma is an indolent low-grade lymphoma derived from marginal zone B cells of MALT that occurs in extranodal organs.^2,3^ MALT lymphoma has been virtually described in all body tissues as it arises from lymphoid populations induced by chronic inflammation in extranodal sites. The most frequently affected organ is the stomach.^4^ However, primary duodenal MALT lymphoma is a very rare neoplasm.^5^ Several aspects of duodenal MALT lymphoma remain to be definitively classified, including its epidemiological characteristics, clinical presentation, association with *Helicobacter pylori*, endoscopic examination findings, histopathological manifestations, and endoscopic ultrasound (EUS) assessments.

Furthermore, the therapeutic approach for this condition warrants further exploration. The importance of this case lies in the concurrent occurrence of gastric and duodenal MALT lymphoma, a rare phenomenon in clinical practice. Consequently, we present a case report describing the presence of primary gastric and duodenal MALT lymphoma in a patient with symptomatic anemia.

## CASE REPORT

In this clinical context, a 56-year-old woman, previously diagnosed with gastroesophageal reflux disease, sought medical attention at the emergency department. She presented with a 1-month history of abdominal pain, primarily localized in the epigastric region, characterized by intermittent, colicky, and intense sensations. In addition, the patient reported experiencing generalized fatigue. Notably, she also reported experiencing associated symptoms of nausea and shortness of breath on exertion. Apart from notable conjunctival pallor during the physical examination, no other remarkable findings were observed.

Laboratory investigation revealed hemoglobin of 6.1 mg/dL. She was transfused with one unit of packed red blood cells. Anemia workup showed iron deficiency, managed with intravenous iron replacement therapy.

**Figure 1.**
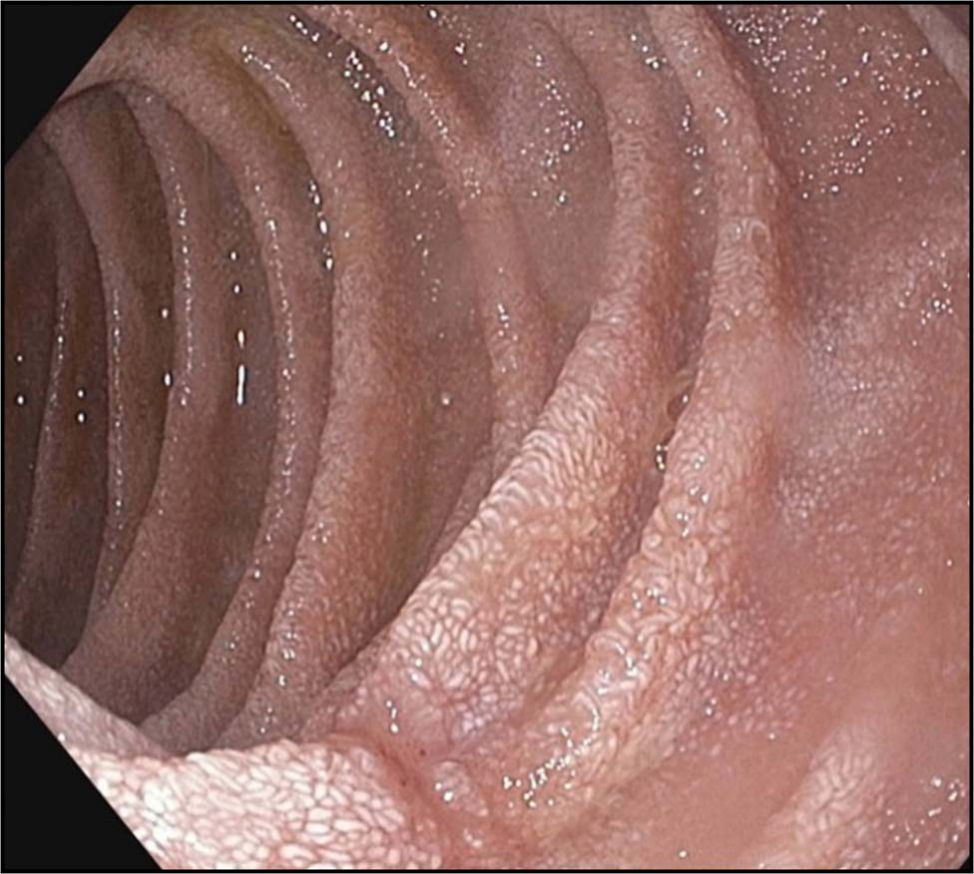
Scattered whitish spots in the second part of duodenum.

The esophagogastroduodenoscopy without endoscopic ultrasound revealed the presence of atrophic gastritis, with multiple whitish spots in the antrum and scattered white duodenal spots, along with erosions in both the first and second parts of the duodenum. Biopsies were obtained for further histopathological examination (Figure 1). On the other hand, colonoscopy was normal.

**Figure 2.**
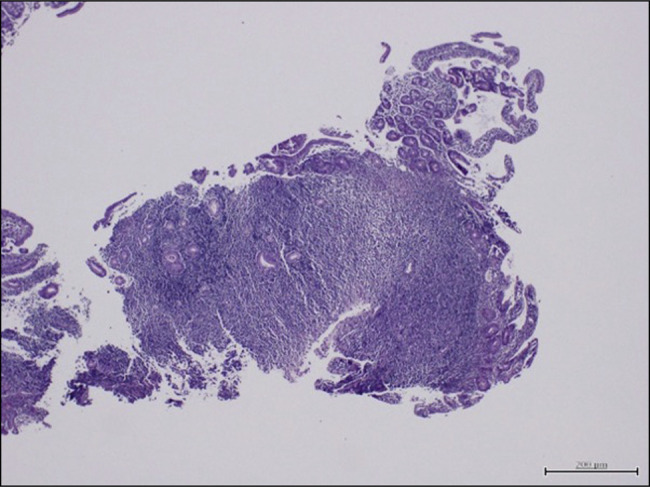
Duodenal biopsy with expanded lamina propria by sheets of atypical lymphocytes (hematoxylin and eosin stain, magnification ×40).

**Figure 3.**
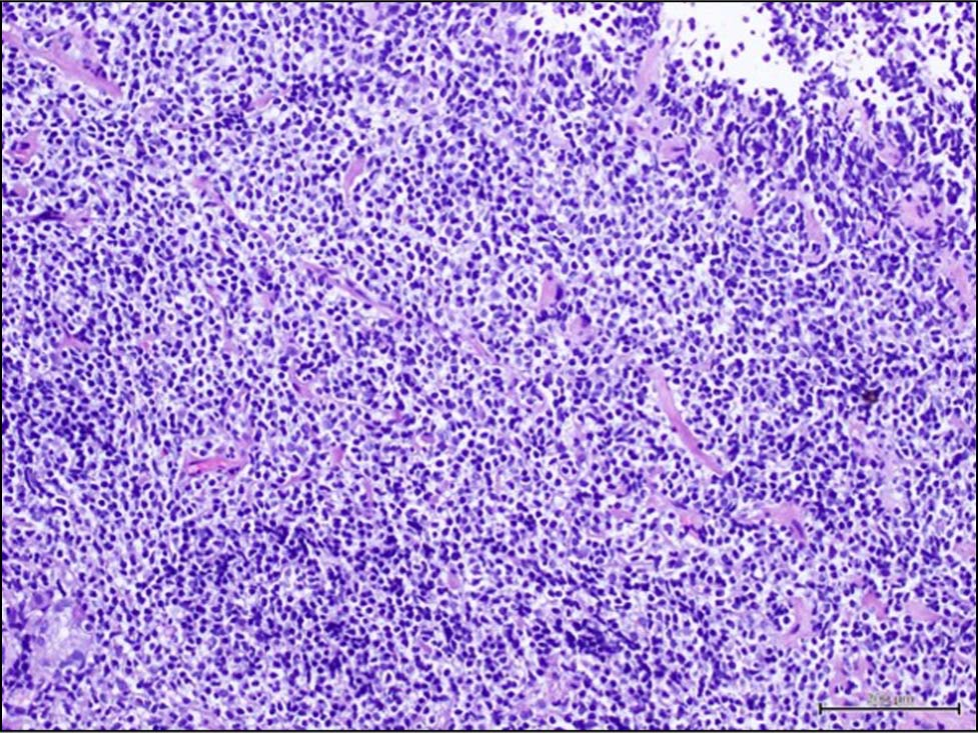
The atypical lymphocytes are small in size with monocytoid appearance (hematoxylin and eosin stain, magnification ×400).

**Figure 4.**
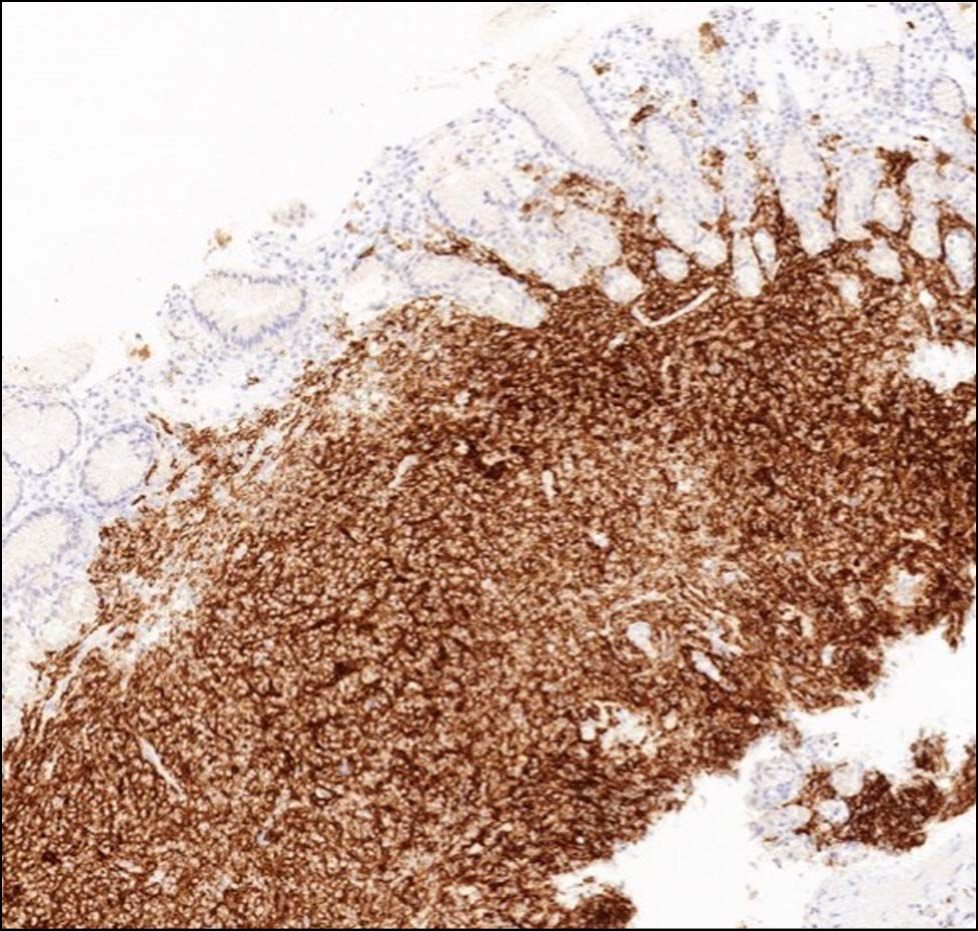
Immunohistochemistry of lymphocytes, positive for CD20 (×100 magnification).

**Figure 5.**
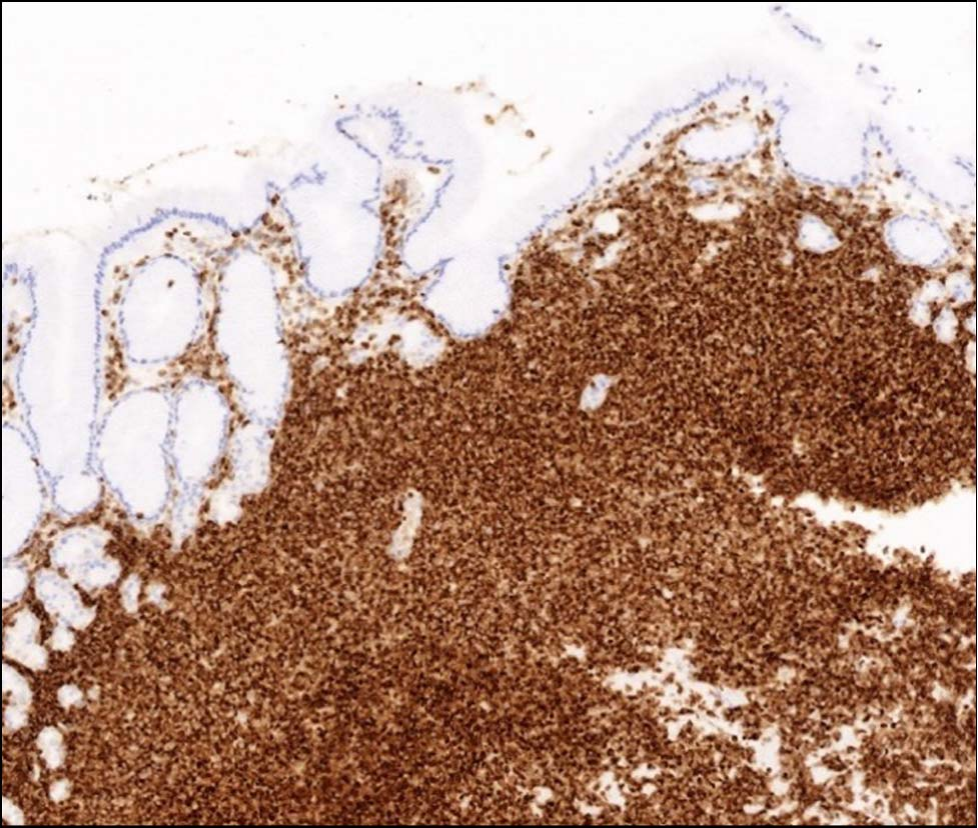
Immunohistochemistry of lymphocytes, positive for bcl-2 (×100 magnification).

**Figure 6.**
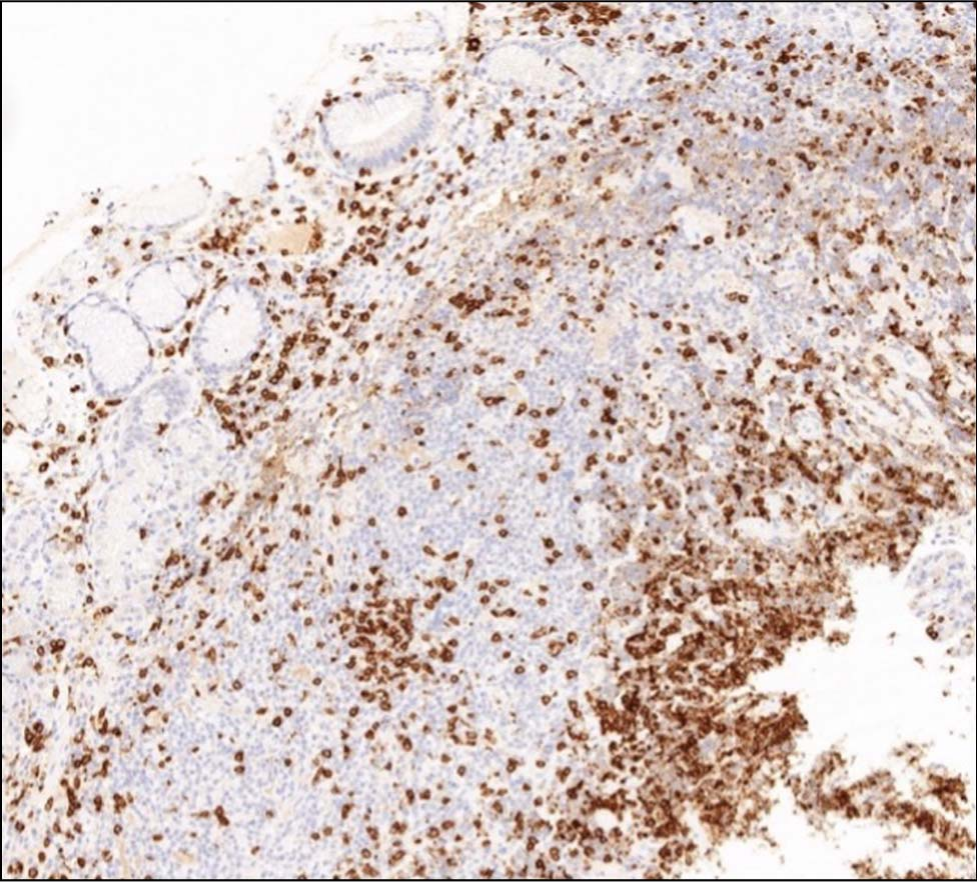
Immunohistochemistry of lymphocytes, negative for CD3 (×100 magnification).

Duodenal biopsy histopathology showed a focal infiltrate of atypical small-sized lymphocytes (Figures 2 and 3). A gastric biopsy showed multiple fragments of gastric mucosa with sheets of small-sized atypical lymphocytes in the submucosa. The infiltrate extends focally to the mucosa and replaces the normal gastric glands. No definite lymphoepithelial lesion is noted. The atypical lymphocytes show a patchy monocytoid appearance. Immunohistochemical studies of biopsies of the atypical lymphocytes were positive for CD20 and bcl-2 while negative for CD3, CD5, CD10, CD43, and bcl-6 (Figures 4–6). CD21 highlights disrupted dendritic meshwork in the background. The proliferation index by ki-67 is low (1%–5%) (Figure 2).

The initial imaging investigations involving contrast-enhanced computed tomography (CT) of the abdomen and pelvis and positron emission tomography (PET) scan demonstrated an absence of lymphadenopathy or any notable hypermetabolic foci.

The patient's treatment regimen commenced with *H. pylori* eradication therapy, consisting of levofloxacin 250 mg twice daily, amoxicillin/clavulanate 1 g twice daily, and omeprazole 20 mg twice daily, all administered orally. Follow-up endoscopy unveiled *H. pylori*-negative and lymphoma-positive biopsies. Subsequently, the patient started on rituximab treatment, and the follow-up endoscopy showed lymphoma-negative biopsies. PET-CT revealed no evidence of hypermetabolic activity, indicating a complete response to therapy.

## DISCUSSION

The stomach is the most common extranodal site for MALT lymphoma. However, duodenal MALT lymphoma is a rare variant, as reported in case reports in the literature. In analyzing the prevalence of duodenal lymphoma forms in the United States, MALT lymphoma comprised around 13.8% of total cases.^6^ The median age for gastric MALT lymphoma is 57 years, although it occurs over a wide age range. It is slightly higher among men than women.^7^ The median age of duodenal MALT lymphoma is 49 years (range 20–72 years). To a small degree, men are more affected than women, as reported in a retrospective study of 13 patients diagnosed with duodenal MALT lymphoma at Asan Medical Center for nearly 10 years from March 1997 to February 2017.^8^

The relationship between *H. pylori* chronic gastritis and gastric MALT lymphoma is well established and understood. More than 90% of cases of gastric MALT lymphoma can be induced by *H. pylori* infection. Moreover, *H. pylori* eradication therapy can lead to complete remission in about 80% of cases of low-grade lymphoma.^9^ The association between duodenal MALT lymphoma and *H. pylori* is still uncertain and lacks conclusive evidence.^10^ Other organisms such as *Campylobacter jejuni* are linked to the development of intestinal MALT lymphoma.^11^ More studies are needed to determine antigens implicated in the pathogenesis of duodenal MALT lymphoma.

The most common site involved in duodenal MALT lymphoma is the bulb, and endoscopically, the most common type is nodular.^8^ Patients with duodenal MALT lymphoma can present with symptoms ranging from no symptoms early in the course of the disease to abdominal pain, anemia, protein-losing enteropathy, and more serious presentations such as gastric outlet obstruction and acute pancreatitis.^5,12–15^

The rarity and variation in the reported cases make the management of duodenal MALT lymphoma challenging. A comprehensive analysis of all duodenal MALT lymphoma case reports, amounting to 26 cases, was documented from 1995 to 2008. No standardized treatment protocol emerged from the reviewed cases, further underscoring the need to elucidate more effective therapeutic strategies to manage this condition.^10^

According to the National Comprehensive Cancer Network guideline for the extranodal nongastric noncutaneous marginal zone B-cell lymphoma, endoscopy with multiple biopsies of anatomical sites by upper endoscopy in the correct clinical vignettes with adequate immunophenotyping by immunohistochemistry and cell surface marker analysis is essential to establish diagnosis. PET/CT scan or CT with contrast has diagnostic quality if systemic therapy is planned. Treatment for localized stages involves site radiation therapy or surgery for specific sites, for selected cases, rituximab, or observation. For extranodal gastric marginal zone lymphoma, in the early stages of the disease, *H. pylori* testing, followed by eradication, is the first step in treatment.

Evaluation of gastric MALT lymphoma by EUS has evolved in importance. It helps in diagnosis and staging, choosing the appropriate treatment, and predicting *H. pylori* eradication therapy response.^16^ However, its role in duodenal MALT lymphoma needs further studies.

Duodenal MALT lymphoma showed higher rates of complications in comparison with gastric MALT lymphoma, such as bleeding, stricturing of the lumen and the transformation to high-grade lymphoma. Such results explain why chemotherapy and radiotherapy should always be considered in addition to eradication therapy.^8^

Conducting a comprehensive review of the newly emerged cases is essential as it aids in enhancing our understanding of the disease and establishing the fundamental principles for effective disease management. Consequently, we present our case to contribute to this field's growing body of knowledge.

## DISCLOSURES

Author contributions: A. Alabed and A. Zayed wrote the paper. O. Sharif and F. Abubaker performed the endoscopy and revised the paper. E. Aljufairi prepared the histopathology slides and participated in writing the paper. A. Alabed is the article guarantor.

Financial disclosure: None to report.

Informed consent was obtained for this case report.
